# Dietary Diversity and Sleep Quality Among Medical Students: Clarifying the Reported Data and Analysis

**DOI:** 10.1002/hsr2.72942

**Published:** 2026-07-29

**Authors:** Muhammad Murtaza, Muhammad Mujtaba Rasool, Ali Jan Yaqubi, Ahmed Asad Raza

**Affiliations:** ^1^ Department of Medicine Services Institute of Medical Sciences Lahore Pakistan; ^2^ Department of Medicine Allama Iqbal Medical College Lahore Pakistan; ^3^ Department of Medicine Kabul University of Medical Science Kabul Afghanistan; ^4^ Department of Medicine Jinnah Sindh Medical University Karachi Pakistan

Dear Editor,

We read with interest the cross‐sectional study by Salam et al., which examined the association between dietary diversity and sleep quality among 246 medical students at Rawalpindi Medical University [1]. The use of a validated sleep assessment and the focus on nutrition in an understudied student population are useful. However, several unresolved inconsistencies and reporting gaps may materially affect the interpretation and reproducibility of the findings.

First, the analytic denominators are inconsistent. Table 1 of the published article, the logistic regression, and the Mann‐Whitney U analysis classify 73 students as having good sleep and 173 as having poor sleep. Table 4 of the published article instead lists 99 and 146, respectively, and totals 245 rather than the stated 246. The corresponding dietary diversity score results also differ: Table 4 of the published article reports *p* = 0.003, whereas the Mann–Whitney analysis reports *p* = 0.047 and the logistic model reports *p* = 0.042. These differences could reflect separate analytic samples or missing observations, but no explanation is provided. More importantly, the medication‐use results appear internally reversed. The text states that 140 women and 75 men used sleep medication; together, these counts equal 215 of 246 participants, or 87.4%, exactly the percentage that Table 2 of the published article labels as using no sleep medication. Table 2 of the published article also describes daily use as rare, and the Discussion states that most participants did not frequently use sleep medication. This is also difficult to reconcile with the exclusion of participants taking medications known to affect sleep. The authors should provide participant accounting for each analysis, explain missing data, verify the Pittsburgh Sleep Quality Index medication coding, and correct the affected tables and analyses. Until then, the sex‐specific medication finding and the related recommendation are not interpretable.

Second, the dietary diversity score is described as an “independent” predictor, but the reported model chi‐square has one degree of freedom, and only dietary diversity is presented as a predictor. Unless covariates were included but not reported, the odds ratio of 0.882 is a crude rather than independently adjusted estimate. This is consequential because body mass index was associated with sleep quality in the same data set, and diet and sleep may share demographic, behavioral, and psychological determinants. STROBE recommends reporting adjusted estimates, the confounders included, and the rationale for their selection; STROBE‐nut further emphasizes transparent reporting of dietary assessment and analytical decisions [2, 3]. A prespecified multivariable model using plausible measured confounders, accompanied by model‐specific sample sizes and sensitivity analyses, would clarify whether the association persists. If key confounders were not measured, the conclusion should be restricted to an unadjusted cross‐sectional association and should not imply that improving dietary diversity will improve sleep.

Third, the Healthy Eating Index analysis cannot be reproduced from the reported methods. The authors do not identify the index version, component definitions, dietary inputs, or scoring algorithm. The Diet Quality Questionnaire is a 29‐item, previous‐day, yes‐or‐no food‐group instrument developed to calculate dietary diversity and other food‐group indicators [4]. In contrast, standard Healthy Eating Index versions contain 13 components, with most scored as intake densities per 1000 kcal and others based on percentages of energy or a fatty‐acid ratio [5]. The article does not explain how these quantitative inputs were obtained from the stated questionnaire. Because Healthy Eating Index values are presented as key secondary results, the authors should report the exact version and component‐level calculation, including the sources of energy and fatty‐acid data. If a nonstandard index was used, it should be named and justified accordingly rather than labeled as the Healthy Eating Index.

We believe these clarifications are necessary before the findings can support institutional nutrition or sleep‐management recommendations. Reconciliation of participant counts, and medication coding, an appropriately reported adjusted analysis, and a reproducible account of the Healthy Eating Index calculation would strengthen the study and define the appropriate limits of its conclusions.

## Author Contributions

**Muhammad Murtaza:** conceptualization, investigation, methodology. **Muhammad Mujtaba Rasool:** validation, visualization. **Ali Jan Yaqubi:** writing – review and editing. **Ahmed Asad Raza:** writing – review and editing, validation.

## Funding

The authors have nothing to report.

## Ethics Statement

Ethics approval was not required because this correspondence did not involve human participants, human data, or animals.

## Conflicts of Interest

The author declares no conflicts of interest.

## Transparency Statement

The corresponding author, Muhammad Murtaza, affirms that this manuscript is an honest, accurate, and transparent account of the study being reported; that no important aspects of the study have been omitted; and that any discrepancies from the study as planned (and, if relevant, registered) have been explained.

## Data Availability

Data sharing is not applicable because no new data were created or analyzed for this correspondence.
